# Visualization of oral function during playing a wind instrument by a lateral dental impression: a proof-of-concept investigation

**DOI:** 10.3389/froh.2025.1522642

**Published:** 2025-05-26

**Authors:** Mariko Hattori, Gen Tanabe, Sebastian B. M. Patzelt, Dirk Schulze, Yuka I. Sumita, Noriyuki Wakabayashi

**Affiliations:** ^1^ Department of Advanced Prosthodontics, Division of Oral Health Sciences, Graduate School of Medical and Dental Sciences, Institute of Science Tokyo, Tokyo, Japan; ^2^ Division of Sports Dentistry, Department of Community Health Sciences, Meikai University School of Dentistry, Sakado, Japan; ^3^ Department of Prosthetic Dentistry, Faculty of Medicine, Center for Dental Medicine, Medical Center - University of Freiburg, University of Freiburg, Freiburg, Germany; ^4^ Private Dental Clinic, Rottweil, Germany; ^5^ Digital Diagnostic Center, Freiburg, Germany; ^6^ Department of Partial and Complete Denture, School of Life Dentistry at Tokyo, The Nippon Dental University, Tokyo, Japan

**Keywords:** dentistry, teeth, wind instrument, brass instrument, dental impression

## Abstract

Embouchure describes the interaction of the teeth, lips, tongue, buccal mucosa, and surrounding muscles when playing woodwind and brass instruments. In dental practice, impression material is used to capture the oral structure and function. In this proof-of-concept investigation, embouchure was examined using a silicone-based dental impression material. The participants were 1 oboe player, 1 alto saxophone player, and 4 tenor saxophone players. Four of the participants were amateurs and 2 were professionals. The dental impression material was mixed and inserted onto the buccal aspect of the upper molars. The musicians blew test tones and maintained embouchure for 30 s while a lateral embouchure impression was taken. The hardened material was removed and scanned using cone-beam computed tomography. The three-dimensional surface data of the impression were exported, and the mean thickness was analyzed. The impressions showed the space between the alveolar and buccal mucosae. The mean thickness ± standard deviation of the impressions was 2.35 ± 0.85 mm. The oboist showed the smallest thickness, while the tenor saxophonists showed the greatest thickness. The method enabled visualization of the unique morphology of each participant. The results suggest that embouchure can be objectively evaluated using the presented technique. Making embouchure impressions to assess oral problems should enable dentists to evaluate changes in music playing resulting from oral problems or for their treatment. Further studies in a larger population are needed to generalize the results.

## 1 Introduction

The teeth and surrounding oral organs play important roles in playing wind and brass instruments (1). It is known that changes in the teeth can lead to differences in music playing (2). It is also known that changes in the oral condition can affect control in music playing (3). It is therefore important to evaluate performance before, during, and after dental treatment to achieve favorable treatment results for musicians.

For example, the sound of music performance is sometimes evaluated during dental treatment such as denture insertion (4), placement of dental implants (5), and the delivery of music splints (6) in order to confirm the effect of the treatment. It is not only important to check the stability of rhythm or the range of sounds, but it is also useful to perform a listening assessment (5).

Embouchure describes the interaction of the teeth, lips, tongue, buccal mucosa, and surrounding muscles when playing woodwind and brass instruments (1). It is known that problems with embouchure can lead to problems in music playing (7). Therefore, it is considered that not only the played sound as an outcome but also the muscle movement and the resulting surface shape should be recorded to ascertain how embouchure might be affected by dental treatment.

Embouchure is examined using various methods, including real-time magnetic resonance imaging (8), electromyography (9), and the use of pressure sensors (10). These methods can provide information on the musical instrument from different viewpoints but the shortcoming is that all of them require special equipment. Therefore, although the method is suitable for research purposes, it may be difficult to apply in the clinical setting.

In dental practice, impression material is often used to capture the oral structure (11). Because the material needs some time to be cured, it is also possible to record the movement of the surrounding tissue by having the patient move while the material is cured (12). In this proof-of-concept investigation, embouchure was examined using a silicone-based dental material.

## 2 Method

**Figure 1:**
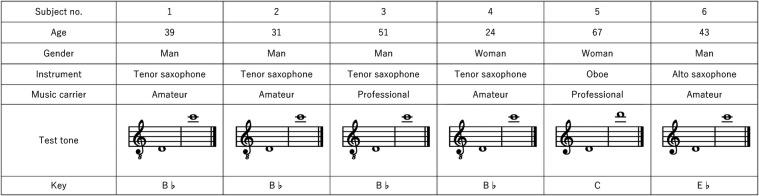
Subjects’ characteristics and the test tones.

The participants were 6 musicians (4 men and 2 women) with a mean age of 42.5 years (Figure 1) and included 1 oboe player, 1 alto saxophone player, and 4 tenor saxophone players. Four of the participants were amateurs and 2 were professionals. The concept of the study was explained verbally and in writing, and written consent was obtained from each participant. The study was approved by the Research Ethics Committee of Tokyo Medical and Dental University (Approval Number: D2016-088).

The participants were seated in a dental chair in an upright position with their musical instrument. A silicone-based dental impression material used for occlusal registration (Exabite II; GC, Tokyo Japan) was inserted with one push of the cartridge dispenser onto the buccal aspect of each upper molar. By mixing the two materials together in the tip, the material hardened due to vulcanization. The participant blew a tone and maintained embouchure for 30 s and then the impression was taken out of the mouth. The procedure was repeated three times for each side as well as for the low- and high-test tones shown in Figure 1.

**Figure 2:**
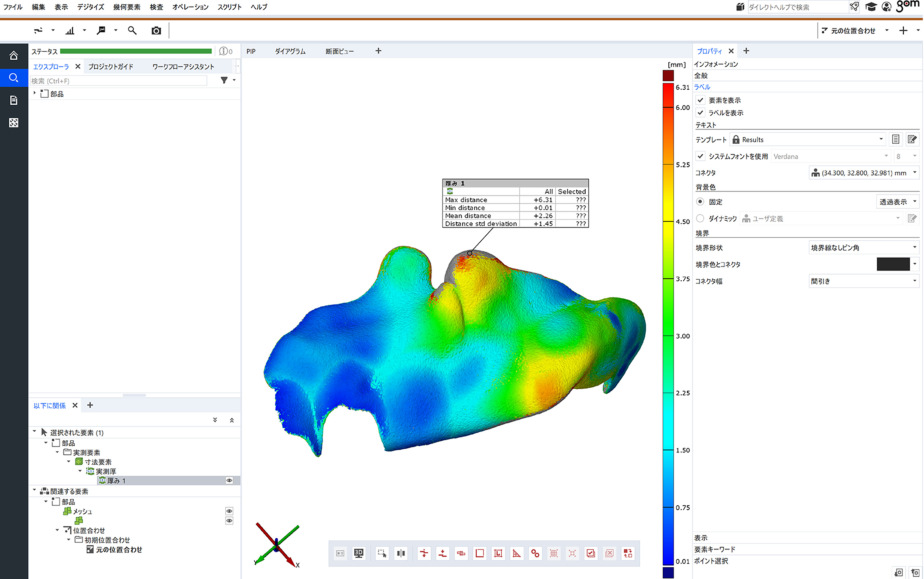
Three-dimensional measurement of an embouchure impression.

After removal, the impression was scanned using cone-beam computed tomography (ProMax 3D Mid; Planmeca, Helsinki, Finland). Surface data of the lateral embouchure impression were exported as a Standard Triangulated Language file, excess parts were trimmed, and the mean thickness was analyzed using three-dimensional (3D) evaluation software (GOM Inspect 2021; GOM, Braunschweig, Germany) (Figure 2). The participant's upper jaw impression was taken with an irreversible hydrocolloid impression material (Algiace Z; Dentsply-Sankin, Tokyo, Japan), and a model was fabricated using dental plaster (New Plastone I; GC). The model was also scanned using cone-beam computed tomography (ProMax 3D Mid), and the surface data were exported as Standard Triangulated Language file. The 3D data were aligned together using 3D evaluation software (GOM Inspect 2021) in order to visualize the relationship between the dentition and the impression.

## 3 Results

**Figure 3:**
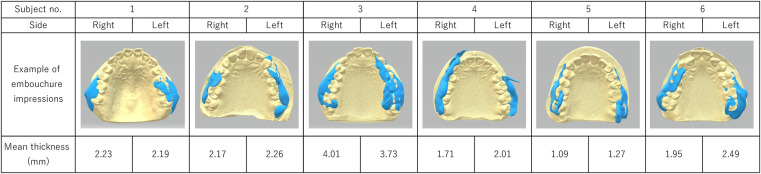
Examples of embouchure impressions and their mean thickness on right- and left-side impressions.

In most cases, the impression was successfully taken; in only a few cases, the material did not stay in position (e.g., it fell down to the lower jaw area) The impressions showed the space between the alveolar and buccal mucosae (Figure 3). The unique morphology of each participant was successfully visualized and was reproducible. In most cases, the impression was thinner in the anterior part and thicker in the posterior part. There was no apparent difference between impressions made when the participants played low and high tones. In some participants the impression was symmetrical, while in others it was asymmetrical. The mean thickness ± standard deviation of the impressions was 2.35 ± 0.85 mm. The oboist showed the smallest thickness (1.18 ± 0.23 mm), while the tenor saxophonists showed the greatest thickness (2.51 ± 0.90 mm).

## 4 Discussion

In this study, intraoral impressions were taken to assess embouchure in musicians. Similar to denture impressions, when the surrounding tissue moves before the material is cured, not only the anatomical structure but also the movement of the tissue is recorded (11). Compared with other methods for observing the oral cavity (8–10), this method is easier because it requires only the general material that every dental office has and does not require any special equipment. Each impression could be taken quickly because the material can be cured in a short time. This suggests that the technique is a suitable method for dentists to evaluate the effect of dental treatment on a musician's embouchure. Because this is a research project, the thickness was examined using a 3D analysis method, but in the clinical setting, simply comparing the morphology might be sufficient for the dentist to grasp a patient's situation.

Some musicians exhibited symmetrical impressions, while others displayed asymmetrical impressions. The observed asymmetry may be attributed to anatomical differences between the two sides or individual playing habits. This finding suggests that clinicians should not aim solely to achieve bilateral symmetry in embouchure impressions. Instead, the focus should be on evaluating changes over time, such as pre- and post-treatment comparisons, rather than comparing the left and right sides.

The standard material for performing occlusal registration was used in the present method. The reason for choosing this material was the short curing time and the ease of handling. Another material considered was the silicone material used for general impressions and impression compounds (11). The silicone material for general impressions has a longer curing time and thus seemed impractical for taking embouchure impressions. Even with the material used in this study, most participants would not be able to continue blowing until the material was cured and would need to maintain embouchure without tone. Thus, it was considered that a regular impression material would not be able to capture an accurate embouchure impression effectively. In contrast, the compound material becomes hard quickly; however, the hardness is temperature dependent and can be difficult to reproduce at a consistent hardness (12). In conclusion, the occlusal registration material was judged suitable for embouchure impression-taking.

Considering the application of emerging technologies, 3D analysis of facial surface morphology during instrument playing could be achieved using facial scanners, which are increasingly utilized in orthognathic and maxillofacial prosthetic research (13). Furthermore, the availability of four-dimensional scanners now enables functional analyses with temporal resolution (14). However, these methods are limited in their ability to capture intraoral dynamics during instrument performance. Real-time magnetic resonance imaging could provide valuable insights into the oro-maxillofacial functional features associated with musical performance (15). Nevertheless, the implementation of such advanced imaging techniques poses significant logistical and financial challenges, making them impractical for routine use in dental clinics catering to musician patients.

In this study, only the space between the molars and the buccal mucosa was evaluated using lateral dental impressions. However, it is conceivable that other anatomical spaces, such as the space between the tongue and the floor of the mouth, could also provide valuable insights into embouchure morphology. Additionally, the limitation of this study to a narrow range of musical instruments represents another shortcoming. Future research will aim to address these limitations and provide a more comprehensive understanding of embouchure characteristics through the use of dental impression materials.

The results of this study suggest that embouchure can be objectively evaluated using the presented technique. The thickness showed that there is more space in the posterior part compared with the anterior part. The reason seems to be that more closure is needed in the front of the mouth to avoid air leakage while more resonance is needed in the back to make sound. However, more detailed research is needed to confirm these assumptions. Furthermore, it was suggested that the morphology of lateral embouchure impressions might differ according to the musical instrument and the individual. Making impressions before and after dental treatment or oral issues occur should enable dentists to evaluate changes in embouchure in musicians. Further studies including a larger number of musicians and a greater variety of instruments are needed to fully evaluate the benefit of the described technique.

## Data Availability

The raw data supporting the conclusions of this article will be made available by the authors, without undue reservation.

## References

[1] Woldendorp KH Boschma H Boonstra AM Arendzen HJ Reneman MF. Fundamentals of embouchure in brass players: towards a definition and clinical assessment. Med Probl Perform Art. (2016) 31:232–43. 10.21091/mppa.2016.4038 27942703

[2] van der Weijden FN Kuitert RB Berkhout FRU van der Weijden GA. Influence of tooth position on wind instrumentalists’ performance and embouchure comfort. J Orofac Orthop. (2018) 79:205–18. 10.1007/s00056-018-0128-2 29532091 PMC5954010

[3] Hattori M Sumita YI Taniguchi H. Influence of changes in the oral cavity on the performance of recorder players: a pilot study. J Prosthet Dent. (2014) 111:425–9. 10.1016/j.prosdent.2013.10.005 24331851

[4] Hattori M Sumita YI Taniguchi H. Sound analysis of a musical performance to evaluate prosthodontic treatment for a clarinet player. J Prosthodont. (2015) 24:71–7. 10.1111/jopr.12166 24920520

[5] Hattori M Patzelt SBM Itoh M Sumita YI Wakabayashi N. Case report: dental treatment for an oboist: post-trauma prosthetic rehabilitation and evaluation of musical performance. Front Psychol. (2023) 13:1022205. 10.3389/FPSYG.2022.1022205 36817383 PMC9930643

[6] Tanabe G Hattori M Obata S Takahashi Y Churei H Nishiyama A Case report: psychoacoustic analysis of a clarinet performance with a custom-made soft lip shield worn to prevent mucosal erosion of lower lip. Front Psychol. (2022) 13:852866. 10.3389/FPSYG.2022.852866 35529561 PMC9069104

[7] Steinmetz A Stang A Kornhuber M Röllinghoff M Delank KS Altenmüller E. From embouchure problems to embouchure dystonia? A survey of self-reported embouchure disorders in 585 professional orchestra brass players. Int Arch Occup Environ Health. (2014) 87:783–92. 10.1007/S00420-013-0923-4 24337629

[8] Hellwig SJ Iltis PW Joseph AA Voit D Frahm J Schoonderwaldt E Tongue involvement in embouchure dystonia: new piloting results using real-time MRI of trumpet players. J Clin Mov Disord. (2019) 6:5. 10.1186/s40734-019-0080-3 31754440 PMC6852982

[9] Franz L Travan L Isola M Marioni G Pozzo R. Facial muscle activity patterns in clarinet players: a key to understanding facial muscle physiology and dysfunction in musicians. Ann Otol Rhinol Laryngol. (2020) 129:1078–87. 10.1177/0003489420931553 32486834

[10] Clemente M Mendes J Moreira A Ferreira AP Amarante JM. A prosthodontic treatment plan for a saxophone player: a conceptual approach. Dent J (Basel). (2018) 6:33. 10.3390/dj6030033 30021940 PMC6162486

[11] Punj A Bompolaki D Garaicoa J. Dental impression materials and techniques. Dent Clin North Am. (2017) 61:779–96. 10.1016/J.CDEN.2017.06.004 28886768

[12] Jassim TK Kareem AE Alloaibi MA. *In vivo* evaluation of the impact of various border molding materials and techniques on the retention of complete maxillary dentures. Dent Med Prob. (2020) 57:191–6. 10.17219/DMP/115104 32649808

[13] Lee D Tanikawa C Yamashiro T. Impairment in facial expression generation in patients with repaired unilateral cleft lip: effects of the physical properties of facial soft tissues. PLoS One. (2021) 16(4):e0249961. 10.1371/journal.pone.0249961 33886591 PMC8061991

[14] Sumita Y Hattori M Semper-Hogg W Gao Y Zhang M Wakabayashi N. Four-dimensional scanning approach to obtain surface data of the face. Int J Maxillofac Prosthetics. (2024) 7(1):6–7. 10.26629/IJMP.2024.03

[15] Schumacher M Schmoor C Plog A Schwarzwald R Taschner CH Echternach M Motor functions in trumpet playing-a real-time MRI analysis. Neuroradiology. (2013) 55(9):1171–81. 10.1007/s00234-013-1218-x 23818231

